# Optic Disc Hemorrhage after Phacoemulsification in Patients with Glaucoma

**DOI:** 10.1155/2014/574054

**Published:** 2014-03-05

**Authors:** Karine D. Bojikian, Daniel B. Moore, Philip P. Chen, Mark A. Slabaugh

**Affiliations:** Department of Ophthalmology, University of Washington, Box 359608, 325 Ninth Avenue, Seattle, WA 98104-2499, USA

## Abstract

*Background*. Optic disk hemorrhage is known to be a risk factor for glaucoma progression. Cataract surgery by phacoemulsification results in large intraocular pressure fluctuations. We aim to investigate whether phacoemulsification is associated with optic disc hemorrhage in patients with glaucoma. *Methods*. This is a retrospective review of consecutive university clinic based glaucoma patients undergoing phacoemulsification alone, with at least 3 visits in the year before and at least 5 visits in the year following phacoemulsification. The presence of optic disk hemorrhage was evaluated with slit lamp biomicroscopy at each clinic visit prior to and following phacoemulsification. *Results*. We evaluated 158 eyes of 158 subjects; 15 (9.5%) had ODH noted at least once during the 2-year study period. Four eyes had ODH identified on postoperative day 1, for a cross-sectional prevalence of 2.5%. Fourteen ODH episodes were noted preoperatively versus 12 episodes postoperatively (*P* = 0.68). Aspirin use was associated with ODH (*P* = 0.015). *Conclusions*. Our cross-sectional study found a prevalence of ODH immediately after CE that was similar to other published rates, and our longitudinal study did not find an increase in ODH in the year after phacoemulsification when compared to the year prior to surgery.

## 1. Introduction

Glaucoma is characterized by retinal ganglion cell degeneration, characteristic changes of the optic disc, and associated visual field loss. Elevated intraocular pressure (IOP) remains the most important known risk factor for the development and progression of glaucoma [1, 2]. Optic disc hemorrhage (ODH) has been associated with glaucoma damage and is considered to be an independent risk factor both for development and progression of the disease [3–5].

The etiology of ODH is poorly understood. Some authors have suggested that it represents rupture of anterior capillaries during posterior bowing of the lamina cribrosa, small infarctions in the capillaries of the optic nerve head, or other poorly defined vascular or connective tissue insults [6]. In spite of its unknown cause, multiple cross-sectional, observational, and prospective studies have identified it as an independent risk factor for progression of glaucomatous visual field loss. As a clinical sign, it remains one of the clearest indicators of optic neuropathy when caring for or identifying individuals with glaucoma.

During cataract extraction by phacoemulsification (CE), high absolute IOP and large IOP fluctuations occur intraoperatively and in the early postoperative period [7, 8]. Although the long-term clinical significance of these short-term IOP fluctuations in glaucoma patients is not clear, these transient high pressures have been documented to cause increased disc cupping and acute reversible visual field depression [9, 10]. To determine whether such large but short-term IOP fluctuations are associated with development of ODH, we examined the cross-sectional prevalence of ODH immediately after CE and the period prevalence of ODH in glaucoma patients undergoing CE.

## 2. Methods

We performed a retrospective review of all glaucoma patients undergoing phacoemulsification as a sole procedure, performed by one surgeon between August 1996 and July 2011 at the University of Washington. This study was approved by the Human Subjects Division of the University of Washington. The diagnosis of glaucoma was based on characteristic optic nerve findings and/or visual field loss, irrespective of IOP. The indication for cataract surgery in all patients was visual acuity that was felt to be reduced by a visually significant cataract. Inclusion criteria for all patients included at least 3 visits in the year prior to scheduled CE and at least 5 visits in the year following CE, including at least 3 visits in the perioperative period (defined as the 6 weeks immediately postoperatively). Exclusion criteria included prior trabeculectomy or other incisional glaucoma surgeries. In cases where both eyes of one patient were eligible, the eye undergoing surgery first was chosen for the study.

Pertinent clinical information prior to and after CE was recorded, as well as intraoperative data. This included baseline demographics, diagnosis subtypes, ophthalmic biometry obtained by IOL Master (Carl Zeiss Meditec Inc., Dublin, CA, USA) or ultrasound A-scan (Innovative Imaging Inc., Ellex, Minneapolis, MN, USA), preoperative IOP (defined as the mean of all clinic visits prior to surgery during the follow-up period), postoperative IOP (defined as the mean of all clinic visits after surgery excluding the six-week perioperative visits), postoperative IOP spike (defined as IOP at least 50% higher than the preoperative IOP during the immediate postoperative period), number of medications, and disease severity indices (visual field mean deviation and pattern standard deviation).

A single glaucoma subspecialist (PPC) examined and treated each of the patients throughout the study period. During each clinic visit, the presence or absence of ODH was specifically determined and noted in the chart using slit lamp biomicroscopy, and, if present, the location was recorded. An ODH was defined as a discrete splinter-shaped area of hemorrhage seen on the optic disc rim, or touching it. Given that ODH has been found to persist for up to 6–8 weeks [8], an ODH was considered new if it recurred at least 2 months later if in the same location, or if it occurred at a different location from a previous ODH. The period prevalence was defined as the proportion of the total population that showed ODH over the time period studied. The incidence rate of ODH was calculated from the number of all detected ODH per patient per year.

Statistical analysis was carried out using PASW Statistics 18.0.0 (IBM SPSS Inc.; New York, NW, USA). Univariate analysis was performed using 2-tailed Student's *t*-test for continuous variables, Pearson chi-square and Fisher's exact test for categorical variables, and Mann-Whitney *U* test for ordinal variables.

## 3. Results

We included 158 eyes of 158 patients who had CE but reviewed notes from both eyes of all patients for notation of ODH to determine incidence per patient and to further examine the relationship between CE and ODH (Table 1). Four eyes had a new ODH noted on postoperative day 1 (POD1), for a cross-sectional prevalence of 2.5%. In another three eyes, an ODH was observed at the same location at the clinic visit preceding CE (range, 2–4 weeks prior to surgery) and on POD1. At postoperative month 1, the ODH persisted in one of these eyes, and in two eyes the ODH seen preoperatively had resolved. Three of four ODH initially identified on POD1 remained at postoperative month 1.

Of the 158 study eyes, fifteen (9.5%) were recorded as having ODH at least once during the two-year study period. Eight of 15 study eyes (53%) had more than one ODH, and a total of 26 new ODH episodes were observed (14 episodes preoperatively versus 12 episodes postoperatively, *P* = 0.68). Four eyes had ODH noted in both the pre- and postoperative year. Eleven of 15 patients (73%) with ODH were using aspirin at the time the ODH was identified (*P* = 0.015); no other significant differences were detected between those with and without ODH for any of the factors examined (Table 2).

We compared the period prevalence of ODH before and after CE (including the eyes with ODH seen on POD 1 as described above). During the one year prior to CE, 9 eyes (5.7%) had ODH noted, compared with 10 eyes (6.3%) during the one year after CE (*P* = 0.81). Notably, significantly more visits occurred during the year after surgery (6.4 ± 1.8 versus 3.9 ± 1.2, *P* < 0.001). We determined the period prevalence of ODH episodes for each 3-month time period before and after CE and found no significant differences between the preoperative and postoperative periods (*P* = 0.37, Table 3).

As an additional comparison and control group, we determined ODH incidence per patient and the ODH period prevalence from the nonsurgical eye. Ten patients (6.3%) had ODH only in the study eye, five patients (3.1%) had bilateral ODH, and two patients (1.2%) had ODH only in the fellow eye (period prevalence of 10.7% in 2 years); the between-eyes correlation of ODH was significant (*R* = .455, *P* < 0.001, Pearson).

## 4. Discussion

Although the precise cause of ODH is still unknown, its prognostic importance for the progression of glaucoma has been well established. The Collaborative Normal Tension Glaucoma Study showed higher rates of progression in glaucoma patients with ODH at baseline, and the Early Manifest Glaucoma Trial found ODH seen during follow-up to be an independent predictor of glaucoma progression, the risk increasing along with increasing ODH frequency [3, 4]. Another clinic-based cohort of 348 glaucoma patients followed for an average of 8.2 years reported twice the rate of visual field progression in eyes that had an ODH at any time during follow-up [5].

Phacoemulsification is performed with a relatively closed infusion and aspiration system, resulting in repeated transiently high IOP and large IOP fluctuations during surgery. Depending on the irrigation fluid bottle height, vacuum, and aspiration settings, IOP frequently exceeds 70 mmHg; however, at times the effective IOP may precipitously drop close to 0 mmHg [7]. During the first 24 hours after CE, up to 40% of glaucoma patients can have a significant increase in IOP [11]. Animal models suggest that this level of IOP fluctuation may cause significant acute laminar deformation in eyes with early to moderate glaucomatous damage [12].

Several studies have attempted to discover factors associated with ODH in glaucoma patients and although some have suggested associations between ODH and hypertension diabetes mellitus, increasing IOP, pseudoexfoliation, and use of aspirin as significant, others could not confirm these findings [13–20]. In the present study, hypertension and diabetes were not associated with ODH, but the use of aspirin was associated with ODH. Given its inhibitory effect on platelet aggregation, aspirin could either increase the risk of developing ODH, could lead to larger ODH that are more likely to be detected, or both. Another possible explanation is that patients using aspirin are more likely to have vascular diseases that may predispose to the development of ODH. The high intereye correlation for ODH that we found is also consistent with the intereye correlation that is known to be present in the progression in glaucoma patients [21].

This study is limited by its retrospective nature. We performed only clinical examination for ODH presence, and this could have led to an underestimation of ODH prevalence. However, presence or absence of ODH was specifically noted for every patient visit and patients were excluded from the study when the optic disc could not be adequately visualized. Also, comparison of studies using solely clinical examination to detect ODH (2.55 to 8.16%/year) versus photography (1.41 to 14.10%/year) versus both modalities (0.22 to 6.88%/year) shows that the period prevalence reported in the literature is similar using different methods [3, 5, 22–25]. Our cross-sectional prevalence rate of new ODH on postoperative day 1 (POD1) was 2.5%, which is comparable with that reported in other cross-sectional studies (2% to 14.1%) [3, 6, 13, 14]. Our period prevalence rate of 10.7% in 2 years (5.35%/year) is also comparable with that reported in other prospective (0.22% to 6.88%/year) and retrospective studies (0.67% to 8.16%/year) of ODH [5, 6, 15–20].

Notably, the additional clinic visits associated with postoperative care resulted in a significantly higher number of clinic visits during the postoperative year compared to the preoperative year. This should result in increased surveillance for ODH and would be expected to bias our findings towards greater detection of ODH during the postoperative year compared to the preoperative year, yet we did not detect any difference from the preoperative visits.

In conclusion, our results imply that the supraphysiologic short-term IOP fluctuation experienced during phacoemulsification does not commonly lead to an increased incidence of ODH in eyes with glaucoma. We hypothesize that disk hemorrhages are more likely to be a result of either vascular events that are not affected by large swings in IOP or are a result of smaller but more chronic changes in lamina cribrosa position.

## Figures and Tables

**Table 1:** Baseline and clinical characteristics of the population studied (*N* = 158), results given as mean ± S.D. where applicable.

Age (years)	74.4 ± 9.74 (range 64–92)
Sex	
Female	96 (61%)
Male	62 (39%)
Race	
Caucasian	124 (78%)
Asian	18 (11%)
African American	12 (8%)
Others	4 (3%)
Glaucoma type	
Primary open angle	87 (56%)
Normal tension	26 (16%)
Pseudoexfoliation	26 (16%)
Chronic angle closure	12 (8%)
Others	7 (4%)
Visual field (*n* = 140)	
Mean deviation (dB)	−5.80 ± 5.88
Pattern standard deviation (dB)	4.51 ± 3.70
Preoperative intraocular pressure (mmHg)	16.7 ± 3.3
Preoperative number of medications	1.9 ± 0.9
Postoperative intraocular pressure (mmHg)	15.1 ± 2.9
Postoperative number of medications	1.9 ± 1.1

**Table 2:** Selected risk factors evaluated between patients who did and did not have an optic disc hemorrhage.

Risk factor	ODH (*n* = 15, 9%)	No ODH (*n* = 143, 91%)	*P*
Age (years)	76.0 ± 7.5	74.2 ± 9.9	0.499
Sex (male)	3 (20%)	59 (41%)	0.109^¶^
Race			0.106^†^
Caucasian	14 (93%)	110 (77%)	
Asian	0	18 (13%)	
African American	0	12 (8%)	
Others	1 (7%)	3 (2%)	
Axial length (mm)	23.79 ± 1.88	24.41 ± 1.95	0.243^§^
Glaucoma type			0.358^†^
Primary open angle	7 (47%)	80 (56%)	
Pseudoexfoliation	5 (33%)	21 (15%)	
Normal tension	1 (7%)	25 (17%)	
Chronic angle closure	2 (13%)	10 (7%)	
Others	0	7 (5%)	
Preoperative visits	3.9 ± 1.3	3.6 ± 0.8	0.454^§^
Postoperative visits	9.1 ± 2.6	9.9 ± 2.1	0.266^§^
Preoperative IOP (mmHg)	15.9 ± 3.1	16.7 ± 3.3	0.332^§^
Postoperative IOP (mmHg)	14.2 ± 2.4	15.2 ± 2.9	0.102^§^
IOP spike	2 (13.3%)	23 (16%)	0.808^†^
Preoperative medications	2.13 ± 1.25	1.92 ± 0.93	0.407^‡^
Postoperative medications	2.13 ± 1.25	1.92 ± 1.1	0.479^‡^
Visual field (dB) (*n* = 140)	(*n* = 14)	(*n* = 125)	
Mean deviation	−6.39 ± 4.56	−5.73 ± 6.03	0.692^§^
Pattern standard deviation	5.18 ± 4.44	4.43 ± 3.62	0.474^§^
CCT (microns) (*n* = 113)	538 ± 25	534 ± 40	0.790^§^
Aspirin use	11 (73%)	58 (40%)	0.015^†^
Hypertension	10 (67%)	102 (71%)	0.705^†^
Diabetes	2 (13%)	32 (22%)	0.417^†^

ODH: optic disc hemorrhage; IOP: intraocular pressure; CCT: central corneal thickness.

^†^Pearson chi-square, ^‡^Mann Whitney *U* test, ^§^2-tail independent sample *t*-test, and ^¶^Fisher's exact test.

**Table 3:** Period prevalence of optic disc hemorrhage episodes for each 3-month time period before and after cataract extraction by phacoemulsification.

Time period	Preoperative period	Postoperative period	*P*
0–3 months	3.2%	3.8%	0.377
4–6 months	3.2%	1.3%	
7–9 months	0.6%	1.9%	
10–12 months	1.9%	0.6%	
